# Posicionamento sobre COVID-19 e Gravidez em Mulheres Cardiopatas – Departamento de Cardiologia da Mulher da Sociedade Brasileira de Cardiologia – 2020

**DOI:** 10.36660/abc.20201063

**Published:** 2020-11-01

**Authors:** Celi Marques-Santos, Walkiria Samuel Avila, Regina Coeli Marques de Carvalho, Alexandre Jorge Gomes de Lucena, Claudia Maria Vilas Freire, Elizabeth Regina Giunco Alexandre, Felipe Favorette Campanharo, Maria Alayde Mendonça R. Rivera, Maria Elizabeth Navegantes Caetano Costa, Marildes Luiza de Castro

**Affiliations:** 1 Secretaria de Saúde do Estado de Sergipe AracajuSE Brasil Secretaria de Saúde do Estado de Sergipe, Aracaju, SE – Brasil; 2 Universidade de são Paulo Faculdade de medicina Hospital das Clínicas São PauloSP Brasil Instituto do Coração (Incor) do Hospital das Clínicas da Faculdade de medicina da Universidade de são Paulo (HCFMUSP), São Paulo, SP – Brasil; 3 Hospital Geral de Fortaleza FortalezaCE Brasil Hospital Geral de Fortaleza, Fortaleza, CE – Brasil; 4 Hospital Agamenon Magalhães RecifePE Brasil Hospital Agamenon Magalhães, Recife, PE – Brasil; 5 Universidade Federal de Minas Gerais Belo HorizonteMG Brasil Universidade Federal de Minas Gerais, Belo Horizonte, MG – Brasil; 6 Hospital do Coração São PauloSP Brasil Hospital do Coração (HCor), São Paulo, SP – Brasil; 7 Hospital Israelita Albert Einstein São PauloSP Brasil Hospital Israelita Albert Einstein, São Paulo, SP – Brasil; 8 Universidade Federal de Alagoas MaceióAL Brasil Universidade Federal de Alagoas, Maceió, AL – Brasil; 9 Cardio Diagnóstico BelémPA Brasil Cardio Diagnóstico, Belém, PA – Brasil; 10 Centro Universitário Metropolitano da Amazônia BelémPA Brasil Centro Universitário Metropolitano da Amazônia (UNIFAMAZ), Belém, PA – Brasil; 11 Centro Universitário do Estado Pará BelémPA Brasil Centro Universitário do Estado Pará (CESUPA), Belém, PA – Brasil; 12 UNIMED BelémPA Brasil UNIMED, Belém, PA – Brasil; 13 Fundação Hospitalar do Estado de Minas Gerais Belo HorizonteMG Brasil Fundação Hospitalar do Estado de Minas Gerais, Belo Horizonte, MG – Brasil

## 1. Contexto

A doença do coronavírus de 2019 (*coronavirus disease* 2019, COVID-19), causada pelo coronavírus da síndrome respiratória aguda grave 2 (*severe acute respiratory syndrome coronavirus* 2, SARS-CoV-2), foi declarada uma pandemia pela Organização Mundial da Saúde (OMS) em 11 de março de 2020.^1^

A COVID-19 é caracterizada pela alta transmissibilidade e pela apresentação variável, de casos assintomáticos ou leves a quadros críticos.^2^ Os sintomas leves da doença incluem tosse seca, dor de garganta, dispneia, manifestações gastrointestinais, fadiga, anosmia e cefaleia, e eventos graves como tromboembolismo e complicações cardiovasculares.^3^

Aproximadamente 10% dos pacientes podem desenvolver pneumonia e progredir para síndrome do desconforto respiratório agudo (SDRA), falência de múltiplos órgãos e óbito.^4^

De fato, evidências epidemiológicas prévias sugeriram fortemente que gestantes apresentaram maior risco de doença grave e óbito por infecções virais durante a pandemia de influenza H1N1^5^ e de dois outros coronavírus patogênicos - SARS (síndrome respiratória aguda grave, *severe acute respiratory syndrome*) e MERS (síndrome respiratório do Oriente Médio, *Middle East respiratory syndrome*).^6^ Por causa disso, a OMS acreditava que gestantes deviam ser consideradas população de risco para a COVID-19.

Posteriormente, o Ministério da Saúde do Brasil incluiu gestantes, puérperas e mulheres que sofreram aborto espontâneo no grupo de alto risco.^7^ Outra questão a se considerar é a alta mortalidade da COVID-19 quando associada a doenças crônicas, tais como cardiopatia, diabetes melito e hipertensão arterial.^8^^,^^9^

Certamente é um desafio manejar uma gestante cardiopata com suspeita ou diagnóstico confirmado de COVID-19, porque a sobreposição de complicações pode aumentar consideravelmente a mortalidade materna.

Portanto, é necessário um Posicionamento sobre COVID-19, Gravidez e Cardiopatia no atual momento de pandemia. Os objetivos deste documento são apresentar aspectos da COVID-19 durante a gravidez e sua concomitância com cardiopatias, além de propor recomendações que possam contribuir para protocolos de assistência a gestantes cardiopatas durante a presente pandemia.

## 2. COVID-19 e Gravidez

### 2.1. Desfecho Materno

De acordo com o conhecimento atual, existem evidências de que gravidez é um fator de risco para COVID-19.^10^ As limitações da experiência mundial tornam difícil estabelecer o desfecho dessa infecção durante a gravidez. Além disso, as diferenças nas políticas de saúde pública e nas condições culturais e socioeconômicas em nível mundial não possibilitam que se chegue a conclusões sobre o prognóstico de gestantes com SARS-CoV-2.^11^

**Figura 1:**
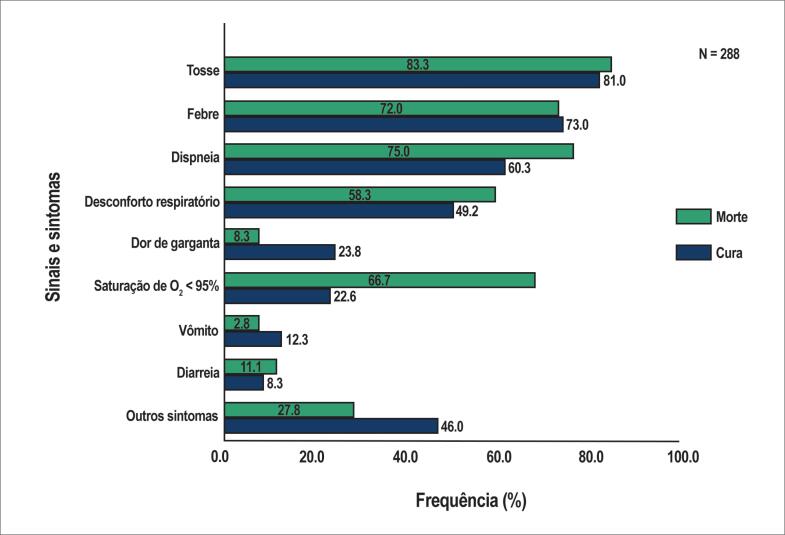
Sintomas e desfecho (cura ou morte) da síndrome do desconforto respiratório por COVID-19 em mulheres grávidas.^13^

Os dados do Ministério da Saúde do Brasil, atualizados em maio de 2020, registraram alta mortalidade em uma coorte de 288 gestantes com SDRA causada pela COVID-19, muitas das quais estavam entre o segundo e o terceiro trimestre de gestação. Houve 36 (12,5%) óbitos maternos, com alta prevalência de cardiopatia entre as comorbidades identificadas nesse grupo (Tabela 1).^12^ Os sinais e sintomas mais frequentes apresentados pelas gestantes com COVID-19 foram semelhantes aos da população geral, e dessaturação de oxigênio foi mais frequente em pacientes que faleceram (Figura 1).^12^

**Tabela 1:** Síndrome do desconforto respiratório por COVID-19 em mulheres grávidas de acordo com a idade gestacional e comorbidades^13^


	Desfecho de mulheres grávidas (N = 288)			
	Cura (n = 252)		Morte (n = 36)	
	n	%	n	%
**Idade gestacional**				
Primeiro trimestre	20	7,9	1	2,8
Segundo trimestre	51	20,2	11	30,6
Terceiro trimestre	168	66,7	22	61,1
Desconhecida	13	5,2	2	5,6
**Comorbidades**				
Doença cardíaca	11	4,4	9	25
Asma	11	4,4	3	8,3
Diabetes mellitus	31	12,3	6	16,7
Hipertensão arterial	10	3,9	5	13,9
Obesidade	11	4,4	4	11,1
Hipotireoidismo	2	0,8	1	2,8
Doença neurológica crônica	3	1,2	0	-
Doença pulmonar crônica	3	1,2	1	2,8
Doença hematológica crônica	9	3,6	0	-
Doença renal crônica	2	0,8	0	-
Imunodeficiência/imunossupressão	3	1,2	0	-

Quanto à atual pandemia de COVID-19, foram levantadas questões sobre a continuação da gravidez, a via de parto ideal, a possibilidade de transmissão viral vertical, isolamento entre os neonatos e as mães, e aconselhamento sobre amamentação.

### 2.2. Desfechos Obstétricos e Fetais

Não existem dados conclusivos que indiquem danos fetais em gestantes com infecção por SARS CoV-2.^14^ Em uma revisão sistemática de 43 casos de COVID-19 durante a gravidez, a única complicação relatada foi uma taxa mais elevada de pré-eclâmpsia e complicações perinatais, tais como parto prematuro.^15^

A via de parto depende de causas obstétricas e da urgência clínica. O parto vaginal não é contraindicado, considerando que não existem evidências convincentes de transmissão vertical. O momento razoável para o parto e a segurança da via de parto devem ser individualizados, não sendo influenciados pela SARS-CoV-2.^16^ Em pacientes críticas, com hipóxia, complicações cardiovasculares ou neurológicas, e sinais de falência progressiva de múltiplos órgãos, o parto por cesariana é o mais apropriado.^17^

### 2.3. Transmissão Perinatal

A expressão do receptor da enzima conversora de angiotensina 2 (ECA2) foi relatada na placenta, particularmente no citotrofoblasto viloso e nos sinciciotrofoblastos. Visto que a ECA2 é altamente expressa nessa região da placenta, isso não apenas aumenta o risco de a mãe contrair SARS-CoV-2 mas também é plausível que ocorra a transmissão de mãe para filho.^10^ Entretanto, a transmissão perinatal de SARS-CoV-2 ainda é controversa.^18^

Dos 75 neonatos de mães com COVID-19, apenas um foi positivo para a doença, e a evolução clínica foi satisfatória, com pequenas alterações nas enzimas hepáticas. Embora alguns bebês negativos para COVID-19 tenham desenvolvido linfocitopenia e apresentado achados radiológicos de pneumonia, além de um caso com coagulação intravascular disseminada, todas as crianças tiveram uma recuperação completa. Com base nesses achados, não é possível descartar que o feto e o recém-nascido possam apresentar uma resposta subclínica à infecção da mãe, e a ocorrência de transmissão viral transplacentária não pode ser descartada.^19^ Portanto, recomenda-se o monitoramento próximo de recém-nascidos de mães infectadas com COVID-19. O leite materno é a melhor fonte de nutrição para os bebês, incluindo aqueles cujas mães têm confirmação ou suspeita de infecção por coronavírus.^20^ Até o momento, o vírus que causa a COVID-19 não foi identificado no leite materno. Uma análise de 114 mães infectadas e seus respectivos recém-nascidos concluiu que o aleitamento não deve ser interrompido. A análise de amostras de leite materno detectou a presença de anticorpos contra o SARS-CoV-2, considerado um fator protetor contra a infecção. Portanto, o aleitamento é recomendado, seguindo as medidas apropriadas de higiene respiratória, de acordo com as recomendações da OMS.^21^ Por outro lado, se a saúde da mãe não permitir o aleitamento direto, o leito materno deve ser coletado previamente e armazenado sem pasteurização. É importante garantir a alimentação do recém-nascido, incluindo a opção de bancos de leite.

Dessa forma, como a experiência com a infecção por SARS-CoV-2 ainda é limitada durante a gravidez, são necessários mais estudos para melhor avaliar os riscos maternos e fetais e os efeitos adversos causados pela COVID-19.

## 3. Gravidez, Cardiopatia e COVID-19

### 3.1. Alterações Fisiológicas da Gravidez que Provocam Complicações Cardiovasculares por COVID-19

Durante a gravidez, alterações nos sistemas imune, respiratório, de coagulação e cardiovascular são os fatores determinantes para o aparecimento de complicações gerais que levam ao óbito materno por todas as causas (Tabela 2). *O sistema imunológico*, durante a gravidez,^22^ desencadeia uma atenuação da imunidade mediada pelas células Th1, devido a uma alteração fisiológica para um ambiente Th2 dominante, o que contribui para o aumento da suscetibilidade materna a patógenos intracelulares e infecções virais, aumentando também a morbidade maternal geral.^23^ As citocinas do tipo Th1 são agentes pró-inflamatórios que contêm interleucinas (IL-1a, IL-1b, IL-6, IL-12) e interferon-gama (IFN-g), enquanto as citocinas do tipo Th2 são agentes anti-inflamatórios que contêm interleucinas (IL-4, IL-10, IL-13) e fator de transformação do crescimento-beta (*transforming growth factor* β, TGF-β). Pacientes com SARS demonstraram uma ativação preferencial da imunidade Th1, resultando em um considerável aumento das citocinas pró-inflamatórias por pelo menos duas semanas após o surgimento da doença que causou danos pulmonares graves.^24^ Entretanto, os pacientes com COVID-19 demonstrem ativação tanto da imunidade Th1 quanto da Th2 durante a evolução da doença, culminando com a presença de IFN-g e IL-1b, além de IL-4 e IL-10. Altos níveis de IL-6 (uma resposta predominantemente Th1) estão associados a um aumento do risco de mortalidade em pacientes com COVID-19.

**Tabela 2:** Impacto das mudanças fisiológicas nos sistemas cardiovascular e respiratório de mulheres grávidas com doenças cardíacas e infecção por SARS-CoV-2

➢ Desregulação do sistema imune materno – atenuação da imunidade mediada por células Th1 e criação de um ambiente de células tipo Th2 – risco de infecções virais
➢ Consumo de oxigênio – hipoventilação, apneia, ou troca gasosa defeituosa – hipoxemia
➢ Capacidade residual funcional reduzida (10% a 25%) – hipoxemia
➢ Hiperemia e edema das vias aéreas superiores – desafios à intubação endotraqueal
➢ Aumento do volume torácico, retardo do esvaziamento gástrico, necessidade de indução em sequência rápida – risco de aspiração
➢ Diminuição da resistência vascular sistêmica – hipotensão, hipoxemia
➢ Aumento da frequência cardíaca e volume sistólico – insuficiência cardíaca
➢ Cuidado na ventilação mecânica Hiperventilação e alcalose – vasoconstrição uterina Hipoventilação e hipercapnia – acidose respiratória fetal PaO_2_ materna deve ser mantida _>_ 70 mmHg – oxigenação fetal
➢ Risco tromboembólico aumentado Aumento de fatores de coagulação (V, VIII, X, e fator de von Willebrand); níveis de proteína S reduzidos Compressão da veia cava inferior e veia ilíaca esquerda pelo útero Trauma local nas veias pélvicas durante o parto; pós-parto de cesariana

*O sistema respiratório*, durante a gravidez, passa por adaptações, devido a influências hormonais e aos efeitos mecânicos do aumento do volume uterino, resultando em uma diminuição progressiva da capacidade pulmonar total e da complacência torácica.^25^ Por esses motivos, a pneumonia por COVID-19 pode ter uma evolução rápida e progressiva, que inicia por consolidação focal, passa por devastação difusa e bilateral do parênquima pulmonar, e culmina em insuficiência respiratória grave. Certamente, a hipóxia materna decorrente da ventilação e da troca gasosa, ambas deficientes, causa redução na perfusão placentária e resulta, em última instância, em sofrimento fetal e até mesmo óbito.

*A ativação do sistema de coagulação*, uma característica da gravidez saudável, ocorre na síntese dos fatores de coagulação II, VII, VIII, IX, e X e do fibrinogênio, assim como a redução dos anticoagulantes endógenos (especialmente antitrombina e proteína S), todos determinantes de um estado de hipercoagulabilidade.^26^ Essas alterações ocorrem progressivamente após o primeiro trimestre da gestação, com a diminuição dos tempos de protrombina, tromboplastina parcial e trombina, favorecendo um enfraquecimento da função anticoagulante. Considerando esses mecanismos, juntamente com a compressão mecânica do plexo venoso nos membros inferiores pelo útero gravídico, justificam uma predisposição ao tromboembolismo durante a gravidez.

*O sistema cardiovascular* sofre uma sobrecarga hemodinâmica que pode agravar o estado funcional de cardiopatias subjacentes. O débito cardíaco aumenta progressivamente no início do primeiro trimestre da gestação, atingindo seu maior aumento no início do terceiro trimestre. Ao mesmo tempo, há uma diminuição da resistência vascular periférica, a qual não não se limita ao plexo uterino e apresenta maior magnitude que a concomitante elevação do débito cardíaco.^27^

### 3.2. Gravidez e Cardiopatia Acarretam Alto Risco para COVID-19

Em função disso, gestantes cardiopatas apresentam maior risco de complicações cardíacas graves, e é obrigatório que a equipe esteja ciente, a fim de se reduzir a maternidade materna nessa coorte de pacientes de alto risco.^28^

No Brasil, a cardiopatia reumática é a principal etiologia de cardiopatias durante a gravidez, seguida de cardiopatias congênitas e cardiomiopatias. Relatos de desfechos maternos demonstraram que cerca de 25% dessas gestantes apresentaram complicações cardiovasculares, incluindo insuficiência cardíaca, tromboembolismo e arritmia, como as causas principais de hospitalização e mortalidade materna.^29^^,^^30^

Vale ressaltar que gestantes com cardiopatias congênitas pertencem a um grupo especial de pacientes, devido à sua grande diversidade de alterações anatômicas e funcionais. Os defeitos cardíacos anatômicos variam de defeitos leves que apresentam o risco usual das gestantes saudáveis a anomalias cardíacas complexas, nas quais o risco é muito alto e até mesmo proibitivo, especialmente na presença de cianose ou hipertensão pulmonar.

Portanto, a estratificação de risco de gravidez para mulheres cardiopatas é essencial, a fim de estimar o prognóstico e planejar as estratégias de prevenção e tratamento para as prováveis complicações.^31^ Nesse sentido, a proposta mais aceita para a estimativa de risco na gravidez é a classificação modificada da OMS, que é dividida em quatro categorias de risco^32^ (Tabela 3).

**Tabela 3:** Classificação do risco cardiovascular materno: Organização Mundial da Saúde, modificada (mOMS)

mOMS I (2,4% - 5%)	mOMS II (5,7% - 10,5%)	mOMS II-III (10% - 19%)	mOMS III (19% - 27%)	mOMS IV (40% - 100%)
Lesões leves ou discretas Estenose pulmonar DSA DSV PCA DAVP Prolapso da válvula mitral Lesões simples operadas Extrassístoles atriais ou ventriculares isoladas	Defeito do septo atrial ou ventricular não operado Tetralogia de Fallot operada Arritmias simples Síndrome de Turner sem dilatação da aorta	Disfunção ventricular leve (FE > 45%) Cardiomiopatia hipertrófica Insuficiência mitral ou aórtica leve a moderada Síndrome de Marfan ou outras doenças hereditárias sem dilatação da aorta Válvula aórtica bicúspide com DAo < 45 mm) Coarctação de aorta operada DSAV	Disfunção ventricular moderada (FE 30%–45%) Cardiomiopatia periparto sem disfunção ventricular Próteses mecânicas Ventrículo direito sistêmico com ou sem disfunção ventricular leve Circulação de Fontan não complicada Cardiopatia congênita cianogênica não operada Outras doenças cardíacas complexas Estenose mitral severa	Hipertensão arterial pulmonar Disfunção ventricular sistêmica (FE < 30% ou classe NYHA III-IV) Cardiomiopatia periparto com disfunção ventricular Lesões obstrutivas graves do coração esquerdo Disfunção ventricular direita grave Dilatação da aorta > 45 mm na síndrome de Marfan, > 50 mm na válvula aórtica bicúspide ou outras doenças hereditárias/síndrome de Turner

Modificado de Balci et al.^32^ DAo: diâmetro da aorta; DAVP: drenagem anômala das veias pulmonares; DSA: defeito do septo atrial; DSAV: defeito do septo atrioventricular; DSV: defeito do septo ventricular; FE: fração de ejeção; NYHA: New York Heart Association; PCA: persistência do canal arterial.

## 4. Sobreposição de Complicações Resultantes de COVID-19, Gravidez e Cardiopatia

### 4.1. Diagnóstico Diferencial

A confirmação do diagnóstico de COVID-19 é essencial. A semelhança das suas manifestações clínicas com as de gestantes cardiopatas pode ocasionar confusão, retardando o diagnóstico de COVID-19 e postergando a implementação de medidas preventivas contra a sua disseminação (Tabela 4).^2^^,^^3^^,^^12^^,^^33^ Por causa disso, considerando a atual epidemia da doença, testes para o SARS-CoV-2 devem ser incluídos como boas práticas, na triagem completa para gestantes cardiopatas.

**Tabela 4:** A tríade COVID-19/doenças cardíacas/gravidez: aspectos e diagnóstico diferencial

	COVID-19	Cardiopatia	Gravidez normal
Sintomas	Febre (> 37,8°C), mialgia, fadiga, anorexia, dor de garganta, congestão nasal e conjuntival, tosse, dispneia, anosmia, ageusia, náusea, vômito, diarreia, dor abdominal	Dispneia/palpitações, dor torácica, síncope, hemoptise, fadiga, inchaço dos membros inferiores, ortopneia, tosse seca	Náusea, vômito, edema/dispneia/fadiga, palpitações, tontura, epistaxe, rinite gestacional, dor de cabeça, dor abdominal
Ocorrência dos sintomas de acordo com a idade gestacional	Qualquer idade gestacional ou puerpério	Geralmente durante o segundo e terceiro trimestre da gravidez ou no puerpério	Qualquer idade gestacional
Histórico	Sem doença cardíaca prévia	Doença cardíaca prévia	Sem doença cardíaca prévia
Aspectos laboratoriais	Teste positivo para COVID-19 de RT-PCR com swab nasofaríngeo Linfocitopenia ALT/AST aumentadas Ureia e creatinina aumentadas Dímero-D aumentado	Altos níveis de BNP	Dímero-D normal ou levemente aumentado
Exames de imagem	Ecocardiograma normal Raio X de tórax com ou sem alterações Tomografia de tórax – opacidade em vidro fosco	Ecocardiograma – lesão cardíaca estrutural Alterações em raio X de tórax/tomografia: cardiomegalia e/ou congestão pulmonar	Ecocardiograma normal Raio X torácico normal

ALT: alanina aminotransferase; AST: aspartato aminotransferase; BNP: peptídeo natriurético tipo B; RT-PCR: reverse transcriptase-polymerase chain reaction.

### 4.2. Impacto da COVID-19 no Sistema Cardiovascular das Gestantes

A atual epidemia de COVID-19 resultou em milhares de óbitos devido a inflamação sistêmica grave e falência de múltiplos órgãos. O sistema cardiovascular também é afetado, resultando em complicações como lesão miocárdica, miocardite, infarto agudo do miocárdio, insuficiência cardíaca, arritmias e eventos tromboembólicos.^34^^,^^35^

A respeito desse tema, uma questão importante a se considerar é o papel fundamental da ECA2 e das complicações cardiovasculares decorrentes da COVID-19.^36^ A ECA2 é uma proteína encontrada na superfície das células epiteliais dos alvéolos pulmonares, consideradas a porta de entrada para o SARS-CoV-2; além disso, a ECA2 quebra a angiotensina II, um fator pró-inflamatório presente no pulmão. Esse desequilíbrio na regulação do sistema imune, aliado ao aumento da demanda metabólica e da atividade pró-coagulante, são responsáveis pelo aumento do risco de desfechos adversos em indivíduos com doença cardiovascular relacionada à COVID-19.^37^

Entretanto, pesquisas recentes sugerem que o vírus também pode causar danos diretos ao coração, através dos receptores de ECA2 presentes no tecido cardíaco.^38^ A prevalência de doenças cardiovasculares em pacientes com COVID-19 ainda não foi determinada com clareza, mas cardiopatias preexistentes podem estar associadas a um desfecho mais grave da COVID-19.

#### 4.2.1. Lesão Miocárdica, Miocardite e Insuficiência Cardíaca

Entre os mecanismos de lesão miocárdica aguda causada pela infecção por SARS-CoV-2, se destacam os seguintes: a expressão da ECA2 no sistema cardiovascular, a tempestade de citocinas desencadeada por um desequilíbrio na resposta autoimune, e a hipoxemia resultante da SDRA.^34^^–^^37^

A inflamação extrema decorrente da COVID-19 pode resultar em lesão endotelial, miocardite e disfunção ventricular, levando a sintomas como dor torácica, dispneia e palpitação.^38^ Essas manifestações podem ser confundidas com as queixas usuais de gestantes cardiopatas, tornando ainda mais difícil o diagnóstico de insuficiência cardíaca durante a gravidez.

Nesse campo, sempre se deve considerar a presença de cardiomiopatia periparto quando a descompensação cardíaca ocorre nos últimos meses da gravidez ou nos meses seguintes ao parto em mulheres anteriormente saudáveis.^39^ A falta de atenção a sintomas como exaustão, dor torácica ou fadiga, que geralmente ocorrem no final da gestação e no pós-parto, pode contribuir para retardar o diagnóstico de cardiomiopatia periparto, e consequentemente, levar a um pior prognóstico e a uma menor chance de recuperação da função miocárdica sistólica. O diagnóstico imediato dessa doença é crucial para a sobrevida do paciente.^40^ Atualmente, os médicos devem estar cientes do diagnóstico diferencial da dispneia relacionada à gravidez, COVID-19 e insuficiência cardíaca da cardiomiopatia periparto para um diagnóstico e uma tomada de decisão inteligentes.^31^^,^^41^ Um algoritmo de manejo especificado e a formação de uma equipe multidisciplinar são fundamentais.

Edema pulmonar também é observado em mulheres saudáveis, uma consequência das alterações substanciais no volume intravascular durante e após o parto. Da mesma forma, alterações hemodinâmicas na gravidez causam um aumento do gradiente através da valva mitral estenótica e congestão pulmonar. Cardiopatia cianótica congênita, lesões por obstrução cardíaca esquerda e disfunção ventricular sistólica grave levam a um aumento do risco. A queda na resistência vascular sistêmica piora a hipoxemia em pacientes com hipertensão pulmonar e cardiopatia congênita complexa.^28^^–^^30^

#### 4.2.2. Estado Hipercoagulável e Eventos Trombóticos

Distúrbios no sistema de coagulação são um aspecto crucial da morbimortalidade na COVID-19. Essa doença vem sendo associada a inflamação e a um estado pró-trombótico, com aumentos da fibrina, dos produtos de degradação da fibrina, do fibrinogênio e do D-dímero.^42^ Nessa situação, pressupõe-se que a combinação de COVID-19, gravidez^43^ e cardiopatias, tais como próstese valvar mecânica ou fibrilação atrial em doenças valvares reumáticas, aumenta consideravelmente o risco de tromboembolismo arterial, requerendo um esquema rigoroso de anticoagulação.^44^^,^^45^

É necessário enfatizar que o D-dímero é um biomarcador pró-trombótico utilizado como critério de exclusão de tromboembolismo pulmonar, mas sua utilidade durante a gravidez apresenta limitações. Os níveis de D-dímero aumentam de forma progressiva e significativa durante a gravidez, atingindo o pico no terceiro trimestre, de forma que é importante considerar que os níveis de D-dímero estão acima do ponto de corte convencional (500μg/L) em 99% das gestantes saudáveis.^46^

Recentemente, o algoritmo YEARS adaptado para a gravidez demonstrou que o diagnóstico de embolia pulmonar durante a gravidez podia ser descartado com segurança na ausência de três parâmetros: 1) sinais clínicos de trombose venosa profunda; 2) hemoptise; 3) embolia pulmonar como o diagnóstico mais provável e D-dímero <1000ng/ml. Entretanto, o ponto de corte para gestantes com COVID-19 ainda é desconhecido. Portanto, a utilização de ferramentas não invasivas como *duplex scan* venoso e exames ecocardiográficos deve ser incentivada para investigar o diagnóstico correto de tromboembolismo ou eventos cardíacos. Eles são amplamente disponíveis, podem ser feitos à beira do leito, têm baixo custo e podem ser repetidos se necessário.^47^

#### 4.2.3. Condição Pró-Inflamatória e Dano Vascular

A inflamação sistêmica e a coagulopatia na COVID-19 aumentam o risco de ruptura de placa aterosclerótica e infarto agudo do miocárdio.^34^^,^^35^ A liberação de citocinas inflamatórias pode causar redução do fluxo sanguíneo coronariano, diminuição do suprimento de oxigênio, desestabilização da placa coronariana e microtrombogênese. A implicação significativa da infecção por SARS-CoV-2 é evidente pela lesão miocárdica aguda com altos níveis de troponina altamente sensível^48^ e/ou novas anomalias no eletrocardiograma e no ecocardiograma, arritmias cardíacas complexas e parada cardíaca. Por outro lado, durante a gravidez, a ocorrência de síndrome coronariana aguda não é comum.^49^ Entretanto, infecções, especialmente no período pós-parto, fazem parte dos fatores de risco para infarto do miocárdio. É importante enfatizar que as causas mais frequentes de infarto do miocárdio durante a gravidez são dissecção espontânea da artéria coronária,^50^ seguida de aterosclerose, trombose coronariana e artérias normais na angiografia com diminuição da microcirculação coronariana. Até agora, não há dados sobre infarto do miocárdio em gestantes com COVID-19.

#### 4.2.4. Condição Pró-Inflamatória, Hipoxemia e Lesão Miocárdica: Indução de Arritmias

Finalmente, arritmias podem estar presentes em pacientes com COVID-19 devido a várias causas simultâneas, tais como inflamação, hipoperfusão, febre ou hipóxia. Por outro lado, mesmo durante uma gravidez normal há distúrbios elétricos cardíacos que causam um aumento da incidência de arritmias cardíacas maternas, variando de batimentos prematuros isolados clinicamente irrelevantes a taquicardias supraventriculares e ventriculares debilitantes.^51^ A ocorrência de arritmias durantes a gestação requer investigação com atenção especial para a identificação ou exclusão de lesão cardíaca estrutural, dano cardíaco elétrico e infecções gerais. Essa prática é fundamental para determinar o tratamento e o prognóstico de arritmias, especialmente aquelas resultantes de efeitos iatrogênicos na pandemia de COVID-19. Sob essas condições, o impacto da terapia para COVID-19 no prolongamento do intervalo QT pode ser verificado pelo Escore de Risco Tisdale (*Tisdale Risk Score*) (https://www.mdcalc.com/tisdale-risk-score-qt-prolongation).^52^

## 5. Resumo e Conclusões

Gravidez em mulheres cardiopatas está incluída no grupo com alto risco de óbito por COVID-19. O conhecimento das complicações concomitantes apresentadas pelas gestantes com COVID-19 permite estabelecer medidas preventivas de acordo com a estratificação de risco para cardiopatias. Portanto, o diagnóstico precoce da infecção por SARS-CoV-2 é fundamental. Nesse cenário, o uso rotineiro do teste de SARS-CoV-2 para o parto é um elemento básico para gestantes cardiopatas. Os benefícios básicos dessas boas práticas incluem a capacidade de identificar a doença precocemente e determinar práticas de isolamento hospitalar, informações sobre cuidados neonatais e orientações sobre o uso de equipamento de proteção individual. Essa rotina proporciona uma oportunidade importante para proteger mães, bebês e profissionais da saúde nesse momento difícil. Certamente os dados a respeito da pandemia de COVID-19 estão em constante evolução e as recomendações seguintes serão revisadas e atualizadas conforme novas informações científicas sejam disponibilizadas.

## 6. Recomendações para Gestante Cardiopatas Durante a Pandemia de COVID-19 (Ver Algoritmo)

➢ Realizar acompanhamento multidisciplinar rigoroso em intervalos curtos de tempo, de acordo com a estratificação de risco da OMS e com as condições maternas e fetais.
➢ Manter os medicamentos prescritos para o tratamento da cardiopatia, com os ajustes necessários da dose, ao longo de toda a gravidez.
➢ Reforçar informações sobre formas de transmissão, sinais e sintomas, e estratégias de prevenção para a COVID-19 durante as consultas pré-natais.
➢ Retirar da linha de frente de contato com pacientes na área de COVID-19 todas as gestantes acima de 24 semanas de gestação.
➢ Entrar em contato com a paciente se ela faltar uma consulta pré-natal agendada.
➢ Aconselhar que a paciente busque atendimento médico em um serviço de referência se tiver qualquer sintoma suspeito de COVID-19 ou apresentar piora no quadro da cardiopatia.
  Realizar investigação padrão para COVID-19 em casos suspeitos, com indicação imediata de hospitalização se houver evidência de comprometimento hemodinâmico e/ou infecção viral grave.
➢ Manter em isolamento por 14 dias, sob monitoramento, pacientes com sintomas leves de COVID-19 e condições cardíacas e obstétricas estáveis.
➢ Indicar hospitalização, na avaliação inicial para suspeita de COVID-19, se a saturação de oxigênio for ≤ 95%, independente da gravidade dos sintomas.
➢ Realizar avaliação criteriosa a respeito de quanto a deterioração clínica é decorrente da COVID-19 e de quanto é decorrente da cardiopatia.
➢ Exames de imagem devem ser realizados, quando indicado, com proteção abdominal, apesar da exposição à radiação.
➢ Tratamento específico para COVID-19 deve ser implementado de acordo com os protocolos estabelecidos para os diferentes estágios da doença.
➢ Testes de reação em cadeia da polimerase via transcriptase reversa (*reverse-transcriptase polymerase chain reaction*, RT-PCR) e devem ser realizados rotineiramente em caso de suspeita de COVID-19 e em todas as pacientes internadas por aborto espontâneo ou 48 horas antes do parto agendado.
➢ Considerar peptídeo natriurético tipo B (*type B natriuretic peptide*, BNP) e fragmento N-terminal do proBNP (*N-terminal fragment of proBNP*, NT-proBNP) como marcadores validados para o diagnóstico de insuficiência cardíaca.
➢ Levar em consideração a influência da gravidez nos níveis de D-dímero como um biomarcador para o diagnóstico de tromboembolismo pulmonar.
➢ Considerar o Posicionamento Brasileiro sobre Cardiopatia e Gravidez no manejo de complicações cardiovasculares.
➢ Avaliar uma possível interação da terapia para COVID-19 com a gravidez em curso, utilizando bancos de dados para medicamentos (www.drugs.com ou www.crediblemeds.org).
➢ Aconselhar o monitoramento próximo de recém-nascidos de mães com COVID-19, visto que a transmissão vertical ainda não pode ser descartada.
➢ Reforçar a amamentação em puérperas com COVID-19, se a saúde da mãe e do recém-nascido permitir, com as seguintes precauções: 1) realizar higiene respiratória durante a amamentação, incluindo o uso de máscara cobrindo a boca e o nariz; e 2) lavar as mãos com sabão e água por 20 segundos antes e depois da amamentação.
➢ Avaliar a possível interação da terapia para COVID-19 com amamentação utilizando bancos de dados para medicamentos (www.drugs.e-lactancia.org);
➢ Sugerir o planejamento de futuras gestações para após o controle da pandemia de COVID-19.

## 7. Algoritmo

**Figure:**
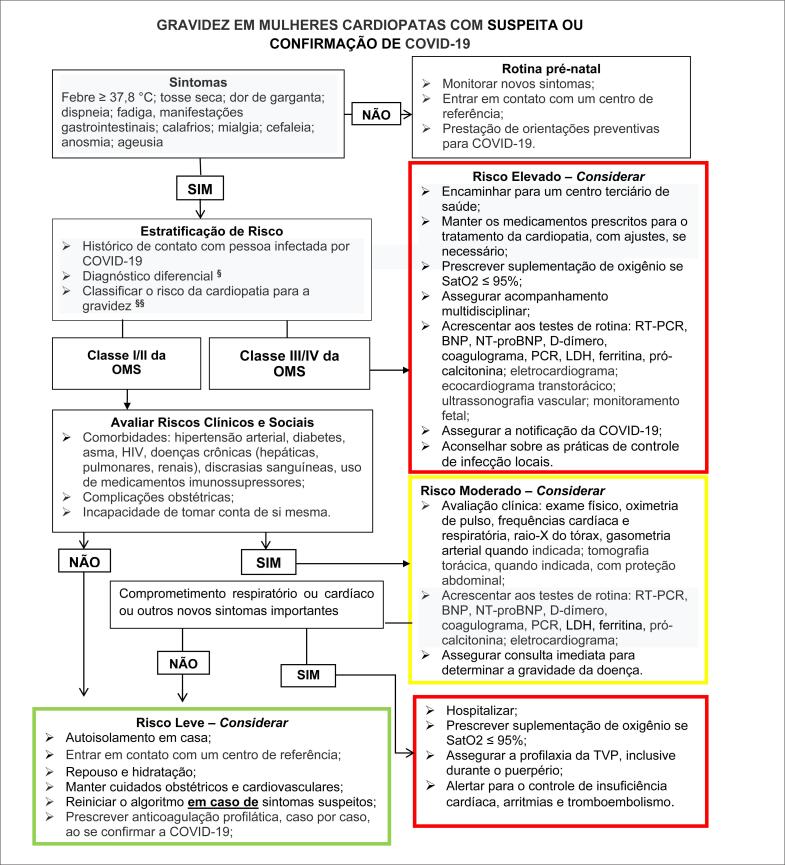
BNP: peptídeo natriurético tipo B (type B natriuretic peptide); LDH: desidrogenas lática (lactic dehydrogenase); NT-proBNP: fragmento N-terminal do proBNP (N-terminal fragment of proBNP); PCR: proteína C-reativa; RT-PCR: reação em cadeia da polimerase via transcriptase reversa (reverse -transcriptase polymerase chain reaction); TVP: trombose venosa profunda; § Tabela 4; §§ Tabela 3.
