# Supplementary material for: Posicionamento sobre COVID-19 e Gravidez em Mulheres Cardiopatas – Departamento de Cardiologia da Mulher da Sociedade Brasileira de Cardiologia – 2020
Source: Arq Bras Cardiol. 2020 Nov 1;115(5):975–86. [Article in Portuguese] doi: 10.36660/abc.20201063 (PMC8452224; doi:10.36660/abc.20201063)
Supplement: Translation

# Translation: Position Statement on COVID-19 and Pregnancy in Women with Heart Disease Department of Women Cardiology of the Brazilian Society of Cardiology – 2020

**Authors:** Celi Marques-Santos, Walkiria Samuel Avila, Regina Coeli Marques de Carvalho, Alexandre Jorge Gomes de Lucena, Claudia Maria Vilas Freire, Elizabeth Regina Giunco Alexandre, Felipe Favorette Campanharo, Maria Alayde Mendonça R. Rivera, Maria Elizabeth Navegantes Caetano Costa, Marildes Luiza de Castro

**Affiliations:** 1 AracajuSE Brazil Secretaria de Saúde do Estado de Sergipe, Aracaju, SE – Brazil; 2 São PauloSP Brazil Instituto do Coração (Incor) do Hospital das Clínicas da Faculdade de medicina da Universidade de são Paulo (HCFMUSP), São Paulo, SP – Brazil; 3 FortalezaCE Brazil Hospital Geral de Fortaleza, Fortaleza, CE – Brazil; 4 RecifePE Brazil Hospital Agamenon Magalhães, Recife, PE – Brazil; 5 Belo HorizonteMG Brazil Universidade Federal de Minas Gerais, Belo Horizonte, MG – Brazil; 6 São PauloSP Brazil Hospital do Coração (HCor), São Paulo, SP – Brazil; 7 São PauloSP Brazil Hospital Israelita Albert Einstein, São Paulo, SP – Brazil; 8 MaceióAL Brazil Universidade Federal de Alagoas, Maceió, AL – Brazil; 9 BelémPA Brazil Cardio Diagnóstico, Belém, PA – Brazil; 10 BelémPA Brazil Centro Universitário Metropolitano da Amazônia (UNIFAMAZ), Belém, PA – Brazil; 11 BelémPA Brazil Centro Universitário do Estado Pará (CESUPA), Belém, PA – Brazil; 12 BelémPA Brazil UNIMED, Belém, PA – Brazil; 13 Belo HorizonteMG Brazil Fundação Hospitalar do Estado de Minas Gerais, Belo Horizonte, MG – Brazil

## 1. Background

The coronavirus disease 2019 (COVID-19), caused by the severe acute respiratory syndrome coronavirus 2 (SARS-CoV-2), was declared a pandemic by the World Health Organization (WHO) on March 11, 2020.^1^

COVID-19 has a high transmissibility and a variable symptom presentation, ranging from asymptomatic or mild symptoms to critical conditions.^2^ Its mild symptoms include dry cough, sore throat, dyspnea, gastrointestinal symptoms, fatigue, anosmia, ageusia, and headache, while serious events include thromboembolism and cardiovascular complications.^3^ Approximately 10% of the patients may present pneumonia and progress to acute respiratory distress syndrome (ARDS), multiple organ failure, and death.^4^

Epidemiological evidence strongly suggested that pregnant women had a higher risk of serious illness and death from viral infections during the previous epidemics of H1N1 influenza^5^ and two other diseases caused by coronaviruses: SARS (severe acute respiratory syndrome) and MERS (Middle East respiratory syndrome).^6^ The WHO thus considered that pregnant women should be considered at risk for COVID-19.

Thereafter, the Brazilian Ministry of Health included pregnant and postpartum women, along with those that had miscarriages, in the high-risk COVID-19 group.^7^ The high mortality of COVID-19 when associated with chronic diseases such as heart disease, diabetes mellitus, and arterial hypertension is also worth noting.^8^^,^^9^

Undoubtedly, a pregnant patient with heart disease who is suspected or confirmed for COVID-19 presents a clinical challenge, since the overlapping of complications could greatly increase maternal mortality.

Therefore, a statement on COVID-19, pregnancy, and heart disease is required at the current moment of this pandemic. The aims of this document are to exhibit aspects of COVID-19 during pregnancy when accompanied by heart diseases and propose recommendations that can contribute to protocols on the care of pregnant women with heart disease during this pandemic.

## 2. COVID-19 and Pregnancy

### 2.1. Maternal Outcome

Current evidence indicates that pregnancy is a risk factor for COVID-19.^10^ Defining an outcome of this infection in pregnant women is difficult due to limitations in case experiences so far. In addition, global differences in public health policies, as well as cultural and socioeconomic conditions, hamper conclusions about the prognosis of pregnant women with SARS-CoV-2.^11^

The Brazilian Ministry of Health, as of May 2020, had registered a high mortality in a cohort of 288 pregnant women with ARDS caused by COVID-19, most of whom were in their second or third trimesters. Data included 36 (12.5%) maternal deaths, with a high prevalence of heart disease among their co-morbidities (Table 1).^12^ The most frequent signs and symptoms presented by pregnant women with COVID-19 were similar to those found in the general population, and oxygen desaturation was more frequent in patients who died (Figure 1).^12^

**Figure 1:** Symptoms and outcome (cure or death) of pregnant women with COVID-19 respiratory distress syndrome.^13^

**Table 1:** Outcome of pregnant women with COVID-19 respiratory distress syndrome according to gestational age and comorbidities^13^


	Outcome of pregnant women (N = 288)			
	Cure (n = 252)		Death (n = 36)	
	n	%	n	%
Gestational age				
First trimester	20	7.9	1	2.8
Second trimester	51	20.2	11	30.6
Third trimester	168	66.7	22	61.1
Unknown	13	5.2	2	5.6
Comorbidities				
Heart disease	11	4.4	9	25
Asthma	11	4.4	3	8.3
Diabetes mellitus	31	12.3	6	16.7
Arterial hypertension	10	3.9	5	13.9
Obesity	11	4.4	4	11.1
Hypothyroidism	2	0.8	1	2.8
Chronic neurological disease	3	1.2	0	-
Chronic pulmonary disease	3	1.2	1	2.8
Chronic hematologic disease	9	3.6	0	-
Chronic kidney disease	2	0.8	0	-
**Immunodeficiency/immunosuppression**	3	1.2	0	-

Considering the current COVID-19 pandemic, questions arise in the care of these patients concerning the continuation of pregnancy, choice of ideal delivery route, possibility of vertical viral transmission, isolation of the neonates, and breastfeeding advisement.

### 2.2. Obstetrical and Fetal Outcomes

No conclusive data currently indicate fetal damage in pregnant woman with SARS-CoV-2 infection.^14^ In a systematic review including 43 pregnant women with COVID-19, the only reported complications were a higher rate of preeclampsia and perinatal complications such as preterm birth.^15^

The route of birth depends on obstetric reasons and clinical urgency. Vaginal delivery is not contraindicated, considering that there is no convincing evidence of vertical transmission. A reasonable labor duration and the safest delivery route should be individualized irrespective of SARS-CoV-2 infection. ^16^ In critically ill parturients (with hypoxia, cardiovascular or neurological complications, or signs of progressive multiple organ failure), a cesarean section is the most appropriate route of birth.^17^

### 2.3. Perinatal Transmission

Expression of the ACE2 receptor has been reported in the placenta, particularly in the villous cytotrophoblast and syncytiotrophoblast cells. This means that the mother could be in higher risk of contracting SARS-CoV-2 and that transmission from mother to child could occur.^10^ However, the perinatal transmission of SARS-CoV-2 is still controversial.^18^

Out of 75 neonates of mothers with COVID-19, only 1 tested positive for the disease; clinical evolution was satisfactory, with slight changes in liver enzymes. Among babies with COVID-19, studies reported lymphocytopenia, radiological findings of pneumonia, and one case of disseminated intravascular coagulation; all children reached full recovery. From these findings, we cannot exclude neither a sub-clinical response to the mother's infection displayed by the fetus and newborn, nor transplacental vertical transmission.^19^ Therefore, close monitoring of newborns whose mothers are infected with COVID-19 is recommended.

Breast milk is the best nutrition source for newborns and infants, including those whose mothers have a confirmed or suspected coronavirus infection.^20^ To date, SARS-CoV-19 has not been detected in breast milk. An analysis of 114 infected mothers and their newborns detected antibodies against SARS-CoV-2 (considered a protective factor against infection) in breast milk and concluded that breastfeeding should not be interrupted. Thus, breastfeeding is recommended, provided that mothers follow appropriate measures of respiratory hygiene and according to WHO recommendations.^21^ On the other hand, if the mother's health does not allow direct breastfeeding, breast milk must be previously collected and kept unpasteurized. Milk banks could be used in order to ensure feeding of the newborn.

Considering that our experience with SARS-CoV-2 infection during pregnancy is still limited, further studies are necessary to better evaluate maternal and fetal risks and the effects of COVID-19 on pregnant patients.

## 3. Pregnancy, Heart Disease, and COVID-19

### 3.1. Physiological Changes of Pregnancy that Induce Cardiovascular Complications by COVID-19

During pregnancy, changes in coagulation and immune, respiratory, and cardiovascular systems are determinant factors that induce complications leading to maternal death from all causes (Table 2). Throughout this period, the immunological system^22^ triggers an attenuation of the immunity mediated by Th1 cells due to a physiological change into a predominantly Th2 environment, which contributes to an increasing maternal susceptibility to intracellular pathogens and virus infections and general maternal morbidity.^23^ The Th1-type cytokines include proinflammatory interleukins (IL-1a, IL-1b, IL-6, IL-12) and interferon-g (IFN-g), while Th2-type cytokines are anti-inflammatory interleukins (IL-4, IL-10, IL-13) and transforming growth factor β (TGF-β). Patients with SARS displayed preferential activation of Th1 immunity, resulting in a marked elevation of proinflammatory cytokines for at least two weeks after disease onset and severe lung damage.^24^ Even though patients with COVID-19 demonstrated activation of both Th1 and Th2 immunities, culminating with the presence of IFN-g and IL-1b, in addition to IL-4 and IL-10, high levels of IL-6 (a predominantly Th1-type response) are associated with an increased risk of mortality.

**Table 2:** Impact of physiological changes in the cardiovascular and respiratory systems of pregnant women with cardiac disease and SARS-CoV-2 infection.

➢ Downregulation of the maternal immune system – attenuation of Th1 cell-mediated immunity to a predominantly Th2 environment - risk of viral infections
➢ Oxygen consumption – hypoventilation, apnea, or impaired gas exchange – hypoxemia
➢ Decreased functional residual capacity (10% to 25%) – hypoxemia
➢ Hyperemia and edema of the upper airways – challenges to endotracheal intubation
➢ ncreased breast volume, delayed gastric emptying, need for rapid sequence induction – risk of aspiration
➢ Decreased systemic vascular resistance – hypotension, hypoxemia
➢ Increased heart rate and stroke volume – heart failure
➢ Caution in mechanical ventilation Hyperventilation and alkalosis – uterine vasoconstriction. Hypoventilation and hypercapnia – fetal respiratory acidosis Maternal PaO_2_ should be kept > 70 mmHg – fetal oxygenation
➢ Increased thromboembolic risks Increase in coagulation factors (V, VIII, X, and von Willebrand factor); decreased protein S levels Compression of the inferior vena cava and left iliac vein by the uterus Local trauma to pelvic veins during delivery; postpartum period of cesarean section

The respiratory system undergoes an adjustment during pregnancy due to hormonal influences and the mechanical effects of increasing uterine volume, resulting in a progressive decrease in total lung capacity and chest wall compliance.^25^ For these reasons, COVID-19 pneumonia may have a rapid and progressive evolution, from focal consolidation to diffuse and bilateral destruction of the pulmonary parenchyma and severe respiratory failure. Cases of maternal hypoxia resulting from impaired ventilation and gas exchange could reduce placental perfusion, ultimately resulting in fetal distress and even death.

The activation of the coagulation system is characteristic of a healthy pregnancy and involves the synthesis of coagulation factors II, VII, VIII, IX, and X, as well as fibrinogen, in addition to a reduction in endogenous anticoagulants (especially antithrombin and protein S); these determine the state of hypercoagulability.^26^ These changes happen progressively after the first trimester with a shortening of prothrombin, partial thromboplastin, and thrombin times that weakens anticoagulant function . When adding to these mechanisms the mechanical compression of the venous plexus on the lower limbs by the gravid uterus, a predisposition to thromboembolism during pregnancy is justified.

The cardiovascular system suffers a hemodynamic overload during pregnancy that may aggravate the functional state of underlying heart diseases. Cardiac output progressively increases in the first trimester, reaching its highest at the beginning of the third trimester. Simultaneously, peripheral vascular resistance (not limited to the uterine plexus) decreases in a greater magnitude than the elevation in cardiac output.^27^

### 3.2. Pregnancy and Heart Disease Bring a High Risk for COVID-19

Since pregnant women with heart disease are at risk for serious cardiac complications, it is mandatory that health care professionals obtain the required knowledge to reduce mortality in this high-risk cohort of patients.^28^

In Brazil, rheumatic heart disease is the main etiology of heart diseases encountered during pregnancy, followed by congenital heart diseases and cardiomyopathies. Reports of maternal outcomes show that around 25% of pregnant women had cardiovascular complications (including heart failure, thromboembolism, and arrhythmias) as the main causes of hospitalization and maternal mortality.^29^^,^^30^

It is worth mentioning that pregnant women with congenital heart disease belong to a special group of patients because of their great diversity in anatomical and functional changes. The anatomical cardiac defects range from mild defects, which do not present additional risk when compared to healthy pregnant women, to complex heart abnormalities that result in very high and even prohibitive risks, especially cyanosis and pulmonary hypertension.

Therefore, a risk stratification of pregnancy in women with heart disease is essential to estimate the prognosis and to plan prevention and treatment strategies for possible complications.^31^ The most accepted risk estimation method proposed for pregnancy is the modified WHO classification, which is divided into four risk categories (Table 3).^32^

**Table 3:** Classification of maternal cardiovascular risk: World Health Organization (mWHO)

mWHO I (2.4% - 5%)	mWHO II (5.7% - 10.5%)	mWHO II-III (10% - 19%)	mWHO III (19% - 27%)	mWHO IV (40% - 100%)
Small or mild lesions Pulmonary stenosis ASD VSD PDA APVD Mitral valve prolapse Simple lesions, successfully operated Isolated atrial or ventricular extrasystoles	Non-operated ASD, VSD Operated tetralogy of Fallot Non-complex arrhythmias Turner syndrome without aortic dilation	Mild ventricular dysfunction (EF > 45%) Hypertrophic cardiomyopathy Mild to moderate mitral or aortic valve disease Marfan syndrome or other inherited diseases without aortic dilation Bicuspid aortic valve with AoD < 45 mm Operated coarctation of the aorta AVSD	Moderate ventricular dysfunction (EF 30%–45%) Peripartum cardiomyopathy without ventricular dysfunction Mechanical prostheses Systemic RV with or without mild ventricular dysfunction Uncomplicated Fontan circulation Non-operated cyanotic heart disease Other complex heart diseases Severe mitral stenosis	Pulmonary arterial hypertension Systemic ventricular dysfunction (EF < 30% or NYHA class III–IV) Peripartum cardiomyopathy with ventricular dysfunction Serious left heart obstructive lesions Severe systemic right ventricular dysfunction Aortic dilation > 45 mm in Marfan syndrome, > 50 mm in bicuspid aortic valve or other inherited diseases / Turner syndrome

Modified from Balci et al.^32^ AoD: aortic diameter; APVD: anomalous pulmonary venous drainage; ASD: atrial septal defects; AVSD: atrioventricular septal defect; EF: ejection fraction; NYHA: New York Heart Association; PDA: patent ductus arteriosus; RV: right ventricle; VSD: ventricular septal defects.

## 4. Overlapping Complications of Covid-19, Pregnancy, and Heart Disease

### 4.1. Differential Diagnosis

Confirmation of COVID-19 diagnosis is essential. The similarity between its clinical characteristics and those of the pregnant woman with heart disease can delay diagnosis and postpone protective measures against its spread (Table 4).^2^^,^^3^^,^^12^^,^^33^ Therefore, considering the current pandemic, tests for SARS-CoV-2 should be included as good practice in the universal screening of pregnant women with heart disease.

**Table 4:** The COVID-19/heart disease/pregnancy triad: features and differential diagnosis

	COVID-19	Cardiopathy	Normal pregnancy
Symptoms	Fever (> 37.8 °C), myalgia, fatigue, anorexia, sore throat, nasal and conjunctival congestions, cough, dyspnea, anosmia, , odynophagia, nausea, vomiting, diarrhea, abdominal pain	Dyspnea/palpitations, chest pain, syncope, hemoptysis, fatigue, lower limb edema, orthopnea, dry cough	Nausea, vomiting, edema/dyspnea/fatigue, palpitations, dizziness, epistaxis, gestational rhinitis, headache, abdominal pain
Occurrence of symptoms according to gestational age	Any gestational age or puerperium	Usually during second and third trimesters of pregnancy or in the puerperium	Any gestational age
History	No previous heart disease	Previous heart disease	No previous heart disease
Laboratory aspects	Positive nasopharyngeal COVID-19 RT-PCR swab test lymphocytopenia Increased ALT/AST Increased urea/creatinine Increased D-dimer	High levels of BNP	Normal or slightly increased D-dimer
Imaging exams	Normal echocardiogram Chest X-ray with or without alterations Chest computed tomography imaging - ground-glass opacity	Echocardiogram - structural cardiac lesion Chest X-ray/computed tomography imaging alterations: cardiomegaly and/or pulmonary congestion	Normal echocardiogram Normal chest-X-ray

ALT: alanine aminotransferase; AST: aspartate aminotransferase; BNP: B-type natriuretic peptide; RT-PCR: reverse transcriptase-polymerase chain reaction.

### 4.2. Impact of COVID-19 on the Cardiovascular System of Pregnant Women

The current COVID-19 pandemic has resulted in thousands of deaths due to severe systemic inflammation and multiple organ failure. The cardiovascular system is also affected by this disease, resulting in complications such as myocardial injury, myocarditis, acute myocardial infarction, heart failure, arrhythmias, and thromboembolic events.^34^^,^^35^

Within this topic, an important subject to be considered is the pivotal role of the angiotensin-converting enzyme 2 (ACE2) in COVID-19 cardiovascular complications.^36^ ACE2 is found on the surface of lung alveolar epithelial cells (considered the entry site for SARS-CoV-2) and catalyzes the cleavage of angiotensin II, a proinflammatory factor. This imbalance in immune regulation, in addition to an increased metabolic demand and procoagulant activity, are responsible for the increased risk of adverse outcomes in patients with COVID-19-related cardiovascular disease.^37^

However, recent research has suggested that the virus may also cause direct damage to the heart utilizing ACE2 receptors in the cardiac tissue.^38^ The prevalence of cardiovascular disease in COVID-19 patients is still unclear, but preexisting heart disease may be associated with more severe COVID-19 outcomes.

#### 4.2.1. Myocardial Injury, Myocarditis, and Heart Failure

Among the mechanisms of acute myocardial injury caused by the SARS-CoV-2 infection, the following stand out: the expression of ACE2 in the cardiovascular system, a cytokine storm triggered by an unbalanced autoimmune response, and hypoxemia resulting from ARDS. ^34^^–^^37^

An extreme inflammatory response to COVID-19 can result in endothelial injury, myocarditis, and ventricular dysfunction, with symptoms of chest pain, dyspnea, and palpitation.^38^ These symptoms overlap with the usual complaints of pregnant women with heart disease, making the diagnosis of heart failure during pregnancy even more difficult.

Peripartum cardiomyopathy should always be considered when cardiac decompensation occurs in the last months of pregnancy or following delivery in previously healthy women.^39^ When symptoms such as exhaustion, chest pain, or fatigue (which usually occur in late pregnancy and postpartum periods) are not given appropriate attention, a delay in the diagnosis of peripartum cardiomyopathy may occur and the patient could have a worse prognosis and less chance of myocardial systolic function recovery. Therefore, prompt diagnosis of this disease is crucial for patient survival^40^ and physicians should be aware of the differential diagnosis of pregnancy-related dyspnea, COVID-19, and heart failure from peripartum cardiomyopathy for efficient decision-making.^31^^,^^41^ A specific algorithm for managing patients and the establishment of a multidisciplinary team is crucial in these cases.

Pulmonary edema is also seen in healthy women as a consequence of major changes in intravascular volume during labor and after delivery. Similarly, hemodynamic changes during pregnancy increase the gradient through the stenotic mitral valve and cause pulmonary congestion. Patients with congenital cyanotic heart disease, left heart obstructive lesions, or severe systolic ventricular dysfunction are at increased risk. A reduction in systemic vascular resistance worsens hypoxemia in patients with pulmonary hypertension and complex congenital heart disease.^28^^–^^30^

#### 4.2.2. Hypercoagulable State and Thrombotic Events

Disorders of the coagulation system are a critical aspect of morbidity and mortality in COVID-19. The disease has been associated with inflammation and a prothrombotic state, with increases in fibrin, fibrin degradation products, fibrinogen, and D-dimer.^42^ In this context, it is assumed that the combination of COVID-19, pregnancy,^43^ and heart conditions such as the use of mechanical valve prostheses or atrial fibrillation in rheumatic mitral valve disease greatly increases the risk of arterial thromboembolism, demanding a rigorous anticoagulation protocol.^44^^,^^45^

Notably, D-dimer is a prothrombotic biomarker used as an exclusion criterion of pulmonary thromboembolism, but its usefulness during pregnancy has limitations. Its levels increase progressively and significantly through pregnancy and peak in the third trimester, making it important to consider that D-dimer levels are above the conventional cut-off (500 μg/L) in 99% of healthy pregnant women.^46^

Recently, the pregnancy-adapted YEARS algorithm proposed that the diagnosis of pulmonary embolism during pregnancy could be safely ruled out in the absence of these three parameters: (1) clinical signs of deep vein thrombosis; (2) hemoptysis; and (3) pulmonary embolism as the most likely diagnosis and D-dimer level < 1000 ng/mL. However, the D-dimer cut-off for pregnant women with COVID-19 is still unknown. Hence, the use of noninvasive tools such as venous duplex scans and echocardiographies is encouraged in the search for the correct diagnosis of thromboembolism or cardiac events. These bedside examinations are highly available, present low costs, and can be repeated if needed.^47^

#### 4.2.3. Proinflammatory Condition and Vascular Damage

The systemic inflammation and coagulopathy exhibited by COVID-19 increase the risk of atherosclerotic plaque rupture and acute myocardial infarction.^34^^,^^35^ The release of inflammatory cytokines can cause a reduction in coronary blood flow and oxygen supply, plaque destabilization, and microthrombogenesis. The significant implication of the SARS-CoV-2 infection becomes evident with acute myocardial injury with high results of highly sensitive troponin assays^48^ and/or new electrocardiogram and echocardiogram abnormalities, complex cardiac arrhythmias, and cardiac arrest. On the other hand, the occurrence of acute coronary syndrome during pregnancy is not common,^49^ even though infections (especially in the postpartum period) are among the risk factors for myocardial infarction. It is important to emphasize that the most frequent causes of myocardial infarction during pregnancy are spontaneous coronary artery dissection,^50^ followed by atherosclerosis, coronary thrombosis, and angiographically normal arteries with impaired coronary microcirculation. So far, no data has been published on myocardial infarctions in pregnant patients with COVID-19.

#### 4.2.4. Proinflammatory Condition, Hypoxemia, and Myocardial Injury Induce Arrhythmias

Finally, arrhythmias may be present in patients with COVID-19, with multiple simultaneous causes such as inflammation, hypoperfusion, fever, or hypoxia. Still, normal pregnancies present electrical cardiac disturbances that increase the incidence of maternal cardiac arrhythmias, ranging from clinically irrelevant isolated premature beats to debilitating supraventricular and ventricular tachycardias.^51^ The occurrence of arrhythmias during pregnancy requires investigation with special attention to identifying or excluding structural cardiac lesions, electric cardiac injury, and general infections. This is a fundamental step when determining the treatment and prognosis of arrhythmias, particularly those with iatrogenic causes amid the COVID-19 pandemic. Under these conditions, the impact of COVID-19 therapy on QT prolongation can be verified on the Tisdale Risk Score (https://www.mdcalc.com/tisdale-risk-score-qt-prolongation).^52^

## 5. Summary and Conclusions

Pregnant women with heart disease are a high-risk group for COVID-19 mortality. The knowledge of overlapping complications between pregnancy and COVID-19 allows the establishment of preventive measures according to cardiac risk stratification. Therefore, early diagnosis of SARS-CoV-2 infection is of crucial importance and the routine use of SARS-CoV-2 testing is fundamental for pregnant women with heart disease . Potential benefits of these good practices include early diagnosis and determination of isolation practices, including guidance on the use of personal protective equipment and neonatal care.

The establishment of a specialized routine provides an important opportunity to protect mothers, babies, and health care professionals during these difficult times. Data on the COVID-19 pandemic are constantly being published and the following recommendations will certainly be reviewed and updated as new scientific information becomes available.

## 6. Recommendations for Pregnant Women with Heart Disease During the COVID-19 Pandemic (See Algorithm)

➢ Maintain a strict multidisciplinary follow-up at short intervals, according to the WHO risk stratification and maternal and fetal conditions;
➢ Maintain the administration of drugs prescribed for the treatment of heart disease, with necessary dose adjustments throughout the pregnancy;
➢ Reinforce information on forms of transmission, signs and symptoms, and prevention strategies for COVID-19 during prenatal visits;
➢ Avoid contact of all pregnant women over 24 weeks with patients in the COVID-19 area;
➢ Contact the patient in case of absence at a scheduled prenatal appointment;
➢ Advise seeking medical care at a referral service if the patient has any suspected COVID-19 symptoms or worsening of heart disease conditions;
➢ Conduct a standard investigation for COVID-19 in suspected cases, with an immediate indication for hospitalization if there is evidence of hemodynamic impairment and/or severity of the viral infection;
➢ Recommend self-isolation and monitoring for 14 days to patients with mild COVID-19 symptoms and stable cardiac and obstetric conditions;
➢ Hospitalization at initial assessment for suspected COVID-19 if O_2_ saturation ≤ 95%, regardless of symptom severity;
➢ Carefully judge the clinical deterioration due to COVID-19 and that resulting from cardiac disease;
➢ Perform imaging tests, when indicated, using abdominal protection to reduce exposure to radiation;
➢ Employ specific treatment for COVID-19 according to established protocols for different stages of the disease;
➢ Routinely perform RT-PCR tests for COVID-19 suspected cases and for all patients on admission for miscarriage or 48 hours before scheduled delivery;
➢ Consider B-type brain natriuretic peptide (BNP) and NT-proBNP as validated markers for the diagnosis of heart failure;
➢ Consider the influence of pregnancy on the D-dimer level as a biomarker for the diagnosis of pulmonary thromboembolism;
➢ Consider the Brazilian Cardiology Society Statement for Management of Pregnancy and Family Planning in Women with Heart Disease in the management of cardiovascular complications;
➢ Evaluate possible interactions between COVID-19 therapy and pregnancy using drug databases (www.drugs.com or www.crediblemeds.org);
➢ Advise close monitoring of newborns of mothers with COVID-19, since vertical transmission is still a possibility;
➢ Reinforce breastfeeding for postpartum women with COVID-19, if the health conditions of the mother and newborn allow it, with the following precautions: (1) The mother should practice respiratory hygiene during breastfeeding, which includes wearing a mask that covers the mouth and nose; and (2) Wash hands with soap and water for 20 seconds before and after breastfeeding;
➢ Evaluate possible interactions between COVID-19 therapy and breastfeeding using drug databases (www.drugs.e-lactancia.org);
➢ Suggest future pregnancy planning considering the control of the COVID-19 pandemic.

## 7. Algorithm

**Figure:** BNP: B-type natriuretic peptide; CRP: C-reactive protein; DVT: deep venous thrombosis; LDH: lactic dehydrogenase; NT-proBNP: N-terminal fragment of proBNP; RT-PCR: reverse transcriptase-polymerase chain reaction; § Table 4; §§ Table 3.
